# Structural Determination and Genetic Identification of the O-Antigen from an *Escherichia coli* Strain, LL004, Representing a Novel Serogroup

**DOI:** 10.3390/ijms222312746

**Published:** 2021-11-25

**Authors:** Jing Wang, Yujuan Xu, Chunjun Qin, Jing Hu, Jian Yin, Xi Guo

**Affiliations:** 1 TEDA Institute of Biological Sciences and Biotechnology, Nankai University, 23 Hongda Street, Tianjin 300457, China; wangjing1998@mail.nankai.edu.cn; 2 Key Laboratory of Carbohydrate Chemistry and Biotechnology, Ministry of Education, School of Biotechnology, Jiangnan University, Lihu Ave. 1800, Wuxi 214122, China; 6200208058@stu.jiangnan.edu.cn (Y.X.); chunjun.qin@jiangnan.edu.cn (C.Q.); jianyin@jiangnan.edu.cn (J.Y.); 3 Wuxi School of Medicine, Jiangnan University, Lihu Ave. 1800, Wuxi 214122, China

**Keywords:** *Escherichia coli*, serogroup, O-antigen, structure, O-antigen gene cluster

## Abstract

The O-antigen is the outermost component of the lipopolysaccharide layer in Gram-negative bacteria, and the variation of O-antigen structure provides the basis for bacterial serological diversity. Here, we determined the O-antigen structure of an *Escherichia coli* strain, LL004, which is totally different from all of the *E. coli* serogroups. The tetrasaccharide repeating unit was determined as →4)-β-d-Gal*p*-(1→3)-β-d-Glc*p*NAc6OAc(~70%)-(1→3)-β-d-Gal*p*A-(1→3)-β-d-Gal*p*NAc-(1→ with monosaccharide analysis and NMR spectra. We also characterized the O-antigen gene cluster of LL004, and sequence analysis showed that it correlated well with the O-antigen structure. Deletion and complementation testing further confirmed its role in O-antigen biosynthesis, and indicated that the O-antigen of LL004 is assembled via the Wzx/Wzy dependent pathway. Our findings, in combination, suggest that LL004 should represent a novel serogroup of *E. coli*.

## 1. Introduction

Lipopolysaccharide (LPS), which is located exclusively in the outermost layer of Gram-negative bacteria, is essential for cell stability and virulence [1]. The LPS molecule typically consists of three components: the lipid A that anchors LPS to the outer membrane, the core oligosaccharide that is a non-repeating oligosaccharide, and the O-antigen (OAg), which is a polymer of repeating oligosaccharide (O-units), each normally being composed of two to seven residues from a broad range of sugars and their derivatives [2].

*Escherichia coli* (*E. coli*) is one of the normal floras of the gastrointestinal tract, but some pathogenic strains of the species may cause a wide range of intestinal and extra-intestinal diseases in humans and animals [3]. To date, more than 180 O serogroups of *E. coli* have been internationally recognized based on the huge variation of its OAg chemical structures [4]. All of the OAg structures have been established and reviewed recently, with 49 different sugars and 19 non-sugar constituents, mainly including acetyl groups, being reported [5].

In *E. coli*, the genes involved in OAg biosynthesis are clustered, namely the O-antigen biosynthesis gene cluster (O-AGC), and, in most cases, maps at a chromosomal locus flanked by two housekeeping genes, *wcaM* encoding colanic acid biosynthesis enzyme and *hisI* encoding phosphoribosyl-AMP cyclohydrolase, respectively [4]. Generally, the O-AGC consists of three main classes of genes: (1) nucleotide sugar precursor synthesis genes, (2) glycosyltransferase genes, and (3) O-unit translocation and polymerization genes (*wzx*/*wzy* in the Wzx/Wzy-dependent pathway and *wzm*/*wzt* in the ABC transporter pathway) [6,7]. In addition to the > 180 serogroups, many O genotypes of *E. coli* strains have been characterized according to their O-AGCs in recent years, very likely representing novel serogroups [8,9,10], however lacking the needed chemical structural data.

Recently, an *E. coli* strain, LL004, was isolated from a human faecal sample in Shandong province, China by us. However, it neither agglutinated against the antisera (IM-EH001, Tianjin Biochip Co., Ltd., Tianjin, China), nor could be tested using Iguchi’s O-genotyping assay targeting almost all well-known *E. coli* serogroups [11]. Here, we established the OAg structure of an *E. coli* strain LL004. Sequence analysis showed that the O-AGC correlated well with the OAg structure of LL004. Moreover, gene knock-out and complementary experiments confirmed that the O-AGC characterized by us is involved in the OAg biosynthesis of LL004. In combination, our results indicate that LL004 may be a novel serogroup of *E. coli*.

## 2. Results and Discussion

### 2.1. Structural Analysis of LL004 OAg

**Figure 1:**
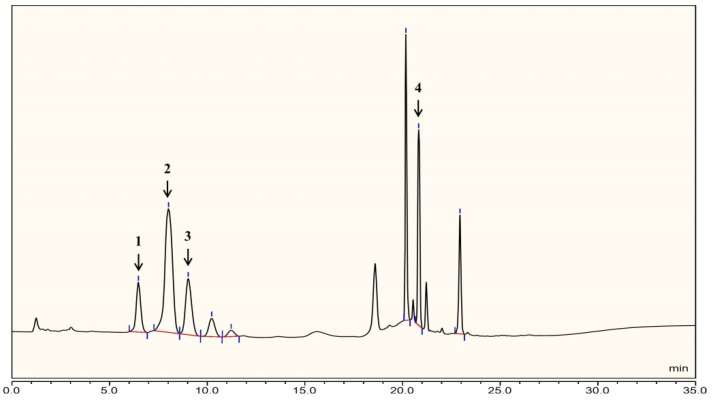
HPLC chromatogram of the hydrolyzed *O*-deacetylated OAg from *E. coli* strain LL004. 1: d-Gal*p*N; 2: d-Glc*p*N; 3: d-Gal*p*; 4: d-Gal*p*A.

The molecular weight of LL004 OAg was determined using high-performance size-exclusion chromatography (HPSEC) which showed a single and symmetrical peak of LL004 OAg, indicating that the average molecular weight of LL004 OAg was 26.1 kDa (Figure S1). Monosaccharide analysis, by the HPAEC-PAD method, of the fully hydrolyzed *O*-deacetylated OAg revealed d-Gal, d-GalN, d-GlcN, and d-GalA (Figure 1). Considering that the *N*-acetyl groups can be removed during the full acid hydrolysis [12], the presence of d-GalNAc and d-GlcNAc should be taken into account.

**Figure 2:**
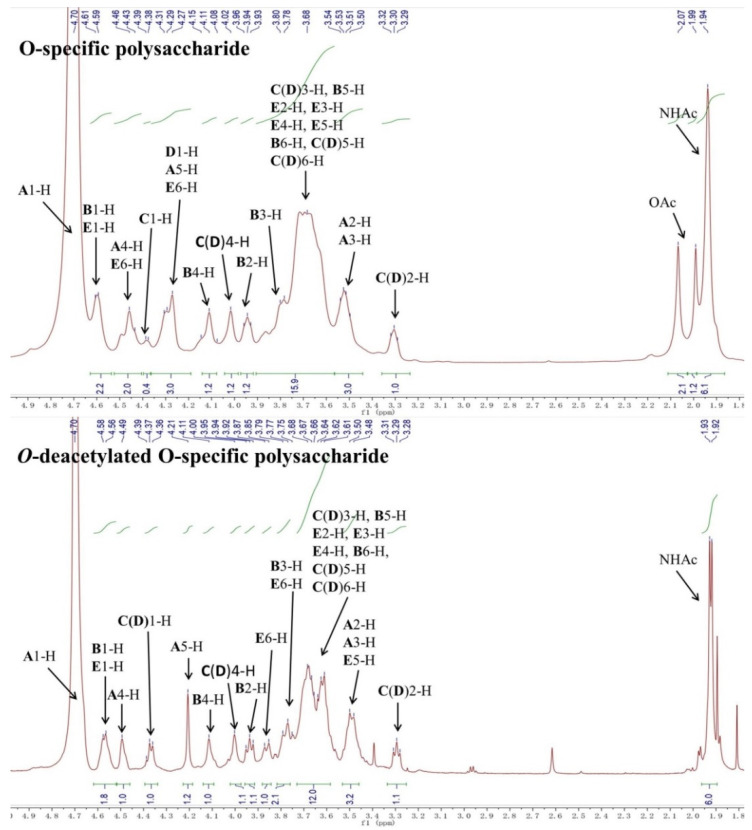
^1^H NMR spectra of the native and *O*-deacetylated O-specific polysaccharide from *E. coli* strain LL004.

**Figure 3:**
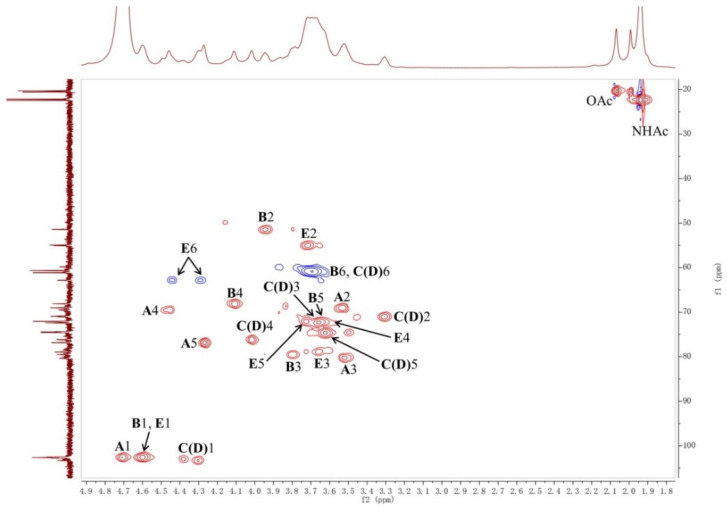
HSQC spectrum of the O-specific polysaccharide from *E. coli* strain LL004.

The LL004 OAg fragment was further analyzed using 1D (^1^H, ^13^C and ^31^P) and 2D (^1^H-^1^H COSY, ^13^C-edited HSQC, coupled HSQC, and ^1^H-^13^C HMBC) NMR experiments. Chemical shift values and integration values obtained with ^1^H NMR spectroscopy showed that the isolation was a homogeneous polysaccharide (Figure 2 and Figure S2). In the upfield region (1.8–2.2 ppm) of the ^1^H NMR spectrum, there were three single sharp signals which correspond to the methyl protons of acetyl groups (Table 1). The integration values of these three peaks indicated that the repeating unit of the polysaccharide was decorated with three acetyl groups. In the downfield region (170–180 ppm) of the ^13^C NMR spectrum, four signals were observed and assigned to the carbonyl group (Figure S3). Another four signals in the upfield region (20–23 ppm) of the ^13^C NMR spectrum were assigned to the methyl carbon of acetyl groups. The comparison between the ^1^H NMR spectra of the OAg and the *O*-deacetylated polysaccharide (Figure 2) indicated that the ^1^H NMR signal at 2.07 ppm corresponded to the methyl protons of the *O*-acetyl group. Furthermore, the ^1^H NMR signal at 1.94 ppm was assigned to the methyl protons of two *N*-acetyl groups. The integration values of the methyl protons of the *O*-acetyl group indicated that the OAg is partially *O*-acetylated. The ^31^P NMR spectrum was dominated by a resonance at 0.5 ppm (Figure S4). The anomeric region (90–110 ppm) of the ^13^C-edited HSQC spectrum contained four signals (Figure 3 and Figure S5). The sugar residues of the repeating unit of the polysaccharide are indicated by capital letters throughout the entire text, tables and figures. The 1-H and 1-C signals of residue **A** appeared at 4.71 and 102.7 ppm, respectively. The 1-H and 1-C signals of residue **B** appeared at 4.60 and 102.6 ppm, respectively. The ^1^H NMR signals at 4.38 and 4.30 ppm were assigned as 1-H of residues **C** and **D**, respectively. The 1-C signals of residues **C** and **D** appeared at 103.0 and 103.3 ppm, respectively. The ^1^H-^1^H COSY spectrum showed connectivities between the signal at 4.60 ppm and two other signals (3.72 and 3.95 ppm) (Figure S6), indicating that the signal at 4.60 ppm corresponded to anomeric protons of two sugar residues. Thus, the 1-H and 1-C signals of residue **E** appeared at 4.61 and 102.6 ppm, respectively. Accordingly, the ^1^H NMR signals at 3.95 and 3.72 ppm were assigned as 2-H of residues **B** and **E**, respectively.

The chemical shifts of all sugar ring protons in residue **A** were assigned using the ^1^H-^1^H COSY spectrum (Figure S6). The 2-H and 3-H signals of residue **A** appeared at 3.55 ppm (^3^*J*_H,H_ 11.0, 6.3 Hz) and 3.52 ppm (^3^*J*_H,H_ 11.0 Hz), respectively. The ^1^H NMR signals at 4.47 and 4.50 ppm were assigned as 4-H of residue **A**. The heterogeneity may be caused by partial *O*-acetylation of OAg. In the COSY spectrum, the signal of 5-H (4.27 ppm) was only connected to the signals of 4-H, indicating the absence of the C6 proton in residue **A**. In the ^1^H-^13^C HMBC spectrum, a connectivity between signals for 5-H (4.27 ppm) and a carbonyl carbon (175.0 ppm) was found, suggesting that the residue **A** contains a 6-carboxyl group (Figure S7). The ^13^C NMR signals of the residue **A** were assigned with the aid of signals of the HSQC spectrum. According to the coupling constants of sugar ring protons, the residue **A** with ^3^*J*_H1,H2_ 6.3 Hz and ^1^*J*_H1,C1_ 160.3 Hz (observed from the coupled HSQC spectrum) (Figure S8) was assigned as a β-d-Gal*p*A. In addition, the chemical shifts of all sugar ring protons and carbons in residue **A** are in agreement with reported NMR data for β-d-Gal*p*A [13].

The chemical shifts of all sugar ring protons in residue **B** were assigned using the ^1^H-^1^H COSY spectrum based on the known chemical shift values of 1-H and 2-H. The 3-H, 4-H, 5-H and 6-CH_2_ signals of residue **B** appeared at 3.80 ppm (^3^*J*_H,H_ 8.9 Hz), 4.12 ppm (singlet), 3.63 ppm, and 3.69 ppm, respectively. The ^13^C NMR signals of the residue **B** were assigned with the aid of signals of the HSQC spectrum. The upfield shift of the 2-C signal (51.4 ppm) indicated the presence of a C-N linkage, suggesting that an acetamido group was located at 2 position of this residue. According to the coupling constants of sugar ring protons, the residue **B** with ^3^*J*_H1,H2_ 8.9 Hz and ^1^*J*_H1,C1_ 165.2 Hz (observed from the coupled HSQC spectrum) was recognized as a β-d-Gal*p*NAc. In addition, the chemical shifts of all sugar ring protons and carbons in residue **B** were in agreement with reported NMR data for β-d-Gal*p*NAc [14].

In the COSY spectrum, the 1-H signals of the residues **C** and **D** were found to correlate with the same proton (3.31 ppm, ^3^*J*_H,H_ 9.0, 6.4 Hz), suggesting that these two 1-H signals belong to the same residue of the polysaccharide. The chemical shifts of all sugar ring protons in residue **C**(**D**) were assigned using the ^1^H-^1^H COSY spectrum. The 3-H, 4-H, 5-H, and 6-CH_2_ signals of residue **C**(**D**) appeared at 3.67 ppm (^3^*J*_H,H_ 9.0 Hz), 4.02 ppm (singlet), 3.62 ppm, and 3.69 ppm, respectively. The ^13^C NMR signals of the residue **C**(**D**) were assigned with the aid of signals of the HSQC spectrum. According to the coupling constants of sugar ring protons, the residue **C**(**D**) with ^3^*J*_H1,H2_ 6.4 Hz and ^1^*J*_H1,C1_ 160.2 Hz (observed from the coupled HSQC spectrum) was assigned as a β-d-Gal*p*. The chemical shifts of all sugar ring protons and carbons in residue **C**(**D**) are in agreement with reported NMR data of β-d-Gal*p* [14]. The slight difference between the chemical shifts of the 1-H in residue **C**(**D**) may be caused by the partial *O*-acetylation of the OAg.

The chemical shifts of all sugar ring protons in residue **E** were assigned using the ^1^H-^1^H COSY spectrum based on the known chemical shift values of 1-H and 2-H. The 3-H, 4-H, 5-H, and 6-CH_2_ signals of residue **E** appeared at 3.64 ppm (^3^*J*_H,H_ 11.0 Hz), 3.65 ppm, 3.73 ppm, and 4.31/4.44 ppm, respectively. The ^13^C NMR signals of the residue **E** were assigned with the aid of signals of the HSQC spectrum. The upfield shift of the 2-C signal (55.1 ppm) indicated the presence of a C-N linkage, suggesting that the residue **E** contains a 2-acetamido group. A significant downfield shift of the 6-H showed that the acetyl group was located at 6 position of residue **E**. Since the coupling constant of 4-H was masked, the monosaccharide type of this residue was confirmed according to the result of the monosaccharide analysis. In addition, except for the 6-H, the chemical shifts of other sugar ring protons and carbons in residue **E** are in agreement with reported NMR data of β-d-Glc*p*NAc [14]. Thus, the residue **E** with ^3^*J*_H1,H2_ 11.0 Hz and ^1^*J*_H1,C1_ 165.2 Hz (observed from the coupled HSQC spectrum) was recognized as a β-d-Glc*p*NAc6OAc.

**Figure 4:**
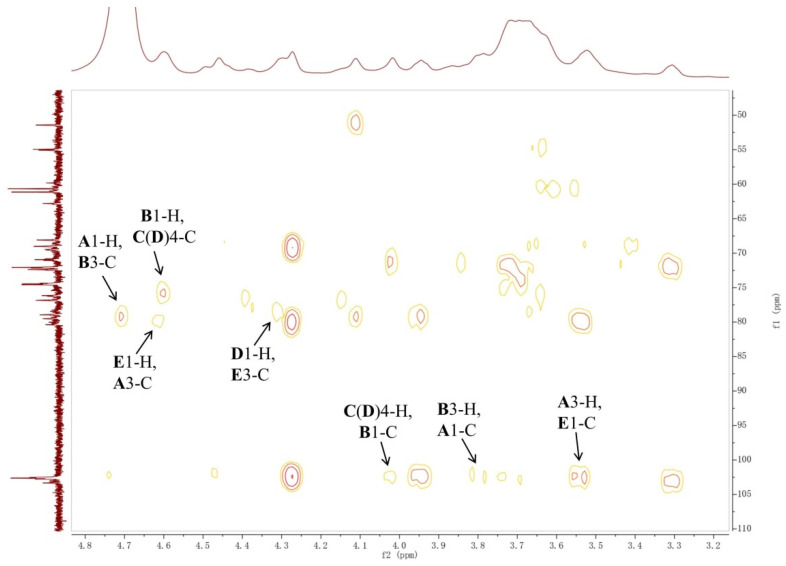
Partial HMBC spectrum of the O-specific polysaccharide from *E. coli* strain LL004.

**Figure 5:**
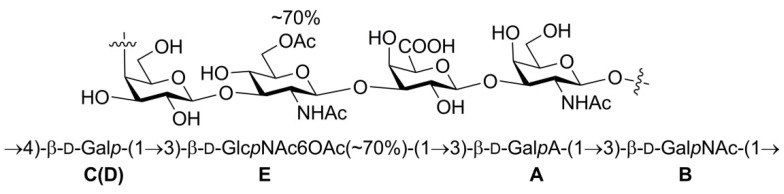
Structure of the O-specific polysaccharide from *E. coli* strain LL004.

Notably, the significant downfield shift of the sugar ring protons were only found for the 6-H in residue **E** associated with the *O*-acetyl group, indicating that there is no other electron withdrawing group, such as phosphate, in the OAg. Although the ^31^P NMR spectrum of the OAg showed a signal at 0.5 ppm, the signal intensity was still low even when a large number of scans (512) at 240 MHz spectrometer frequency was used. In addition, no signal was observed in the ^31^P NMR spectrum of the *O*-deacetylated polysaccharide obtained under the same NMR experiment. Thus, the ^31^P NMR signal of the OAg may be correlated with a very small amount of fragment from LPS. The sequence of sugar residues in the polysaccharide was identified by the HMBC spectrum (Figure 4 and Figure S7). The cross-peaks between the 1-H of residue **A** and the 3-C of residue **B**, the 1-C of residue **A,** and the 3-H of residue **B** were observed, indicating the connectivity between the anomeric position of residue **A** and the 3 position of residue **B**. The correlations of the 1-H of residue **B** and the 4-C of residue **C**(**D**), the 1-C of residue **B,** and the 4-H of residue **C**(**D**), revealed that the residue **B** was linked to the 4 position of the residue **C**(**D**). The cross-peaks between the 1-H of residue **D** and 3-C of residue **E** was observed, suggesting that the residue **D** was connected to the 3 position of residue **E**. The connectivity between the anomeric position of residue **E** and the 3 position of residue **A** was confirmed by the cross-peaks between the 1-H of residue **E** and the 3-C of residue **A**, the 1-C of residue **E,** and the 3-H of residue **A**. It was concluded that the polysaccharide is composed of the tetrasaccharide repeating unit: →4)-β-d-Gal*p*-(1→3)-β-d-Glc*p*NAc6OAc-(1→3)-β-d-Gal*p*A-(1→3)-β-d-Gal*p*NAc-(1→(Figure 5).The slight differences between the chemical shifts of the residue **C**(**D**) 1-H and the residue **A** 4-H may have resulted from the partial *O*-acetylation of the residue **E**. The NMR data of the *O*-deacetylated polysaccharide (Table 2, Figure 2 and Figures S9–S11) are in agreement with the proposed tetrasaccharide. The replacements of the ^1^H NMR signals of residue **E** 6-H from 4.31/4.44 ppm to 3.75/3.86 ppm further confirmed the *O*-acetylation of this 6-OH. According to the integration value of the methyl protons (2.07 ppm) of the *O*-acetyl group in the ^1^H NMR spectrum of the OAg (Figure 2), the ratio of 6-*O*-acetylation in residue **E** is about 70%.

### 2.2. LL004 O-AGC Is Correlated Well to the OAg Structure

**Figure 6:**
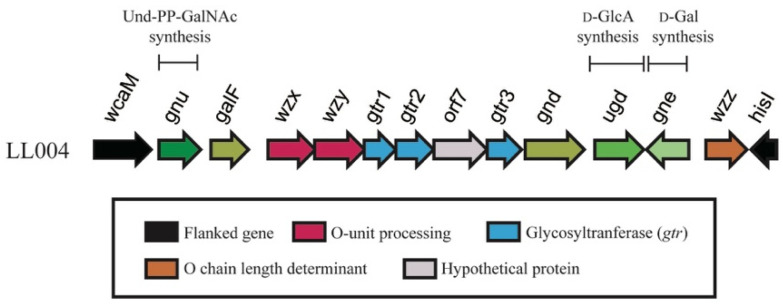
The O-antigen gene cluster of LL004, with the color scheme being shown below.

The O-AGC region of LL004 is located between *wcaM* and *hisI* genes, and consists of 12 *orfs* (Figure 6). Most genes for O-antigen biosynthesis are directly flanked by two housekeeping genes, *orf2* (*galF*) and *orf9* (*gnd*), while orf10 (*ugd*) and *orf12* (*wzz*) are located between *gnd* and *hisI*, as the case in most *E. coli* serogroups. Characteristics of all *orfs* within LL004 O-AGC are summarized in Table 3.

In the LL004 O-unit, there is one residue each of d-GalNAc, d-GlcNAc, d-Gal, and d-GalA. In most *E. coli* serogroups, the first sugar of an O-unit is either d-GlcNAc or d-GalNAc. WecA, encoded by the *wecA* gene within the enterobacterial common antigen (ECA) gene cluster, is always the initial transferase which transfers a GlcNAc-P residue from UDP-GlcNAc to Undecaprenyl phosphate (Und-P), thus forming Und-PP-GlcNAC to initiate the OAg biosynthesis [15]. The Gnu epimerase, with its gene located upstream of *galF*, converts Und-PP-GlcNAc to Und-PP-GalNAc when d-GalNAc is the initial sugar [16]. Thus, *orf1* was assigned *gnu* based on the BLAST research and its product should be involved in the first d-GalNAc residue biosynthesis, i.e., Und-PP-GalNAc formation. *orf11* was assigned *gne* by us, whose product catalyzes the isomerization from UDP-d-GlcNAc to UDP-d-GalNAc or from UDP-d-Glc to UDP-d-Gal [17]. Therefore, it is proposed that Orf11 is responsible for the biosynthesis of the d-Gal residue of LL004 O-unit. In addition, *orf10* was assigned *ugd*, which encodes the UDP-glucose 6-dehydrogenase. The function of Ugd has been identified for the formation of UDP-d-GlcA from UDP-d-Glc [18]. We therefore deduce that the enzyme encoded by *orf10* is involved in UDP-d-GlcA biosynthesis. The formation of UDP-d-GalA from UDP-d-GlcA is catalyzed by UDP-galacturonatenase (Gla), whose gene, however, always maps elsewhere on the chromosome [19]. As d-GlcNAc is a common sugar in bacteria, and the gene for synthesis of its nucleotide precursor is also usually located outside the O-AGC [19], the gene for LL004 d-GlcNAc biosynthesis was not observed in LL004 O-AGC.

Three glycosyltranferase genes (*orf5*, *6* and *8*) were annotated in the LL004 O-AGC, and the enzymes encoded by them are expected for the assembly of the tetrasaccharide O-unit. Orf8 shares 29% identity level (79% coverage) with the galactosyltransferase WbgO [GenBank accession AF461121], which has been identified in the formation of the β-d-Gal-(1→3)-d-GlcNAc linkage of *E. coli* O55 [20]. As this is the only common linkage between LL004 and *E. coli* O55, it is proposed that Orf8 is also involved in β-d-Gal-(1→3)-d-GlcNAc formation in LL004. Another glycosyltranferase, Orf6, shares 22% identity level (44% coverage) with the glycosyltranferase WfbZ of *E. coli* O147 [GenBank accession DQ868766], and β-d-GalA-(1→3)-d-GalNAc is the only common linkage between the O-units of LL004 and O147 [21]. Therefore, it is suggested that Orf6 is responsible for the transfer of UDP-d-GalA to d-GalNAc via β1→3 linkage in LL004. Moreover, based on the above analysis, it is reasonable to predict that the remaining glycosyltranferase, Orf5, catalyzes the formation of the β-d-GlcNAc-(1→3)-d-GalA linkage.

*orf3* and *orf4* were assigned *wzx* and *wzy*, respectively, meaning that it is very likely that LL004 synthesizes its OAg via the Wzx/Wzy dependent pathway. *orf12* was annotated as *wzz*, whose product is the OAg chain length determinant [22]. The function of the remaining gene, *orf7*, could not be resolved via BLAST research, and has, therefore, been assigned as a hypothetical protein encoding gene. Our chemical data showed that the d-GlcNAc residue residue is modified by an acetyl group, however, no acetyl transferase gene was found in the LL004 O-AGC. It is proposed that the relevant gene must be located elsewhere at the chromosome.

Overall, the O-AGC of LL004 is fully consistent with the OAg structure.

### 2.3. Deletion and Complementation Testing Confirmed the Functionality of the LL004 O-AGC

**Figure 7:**
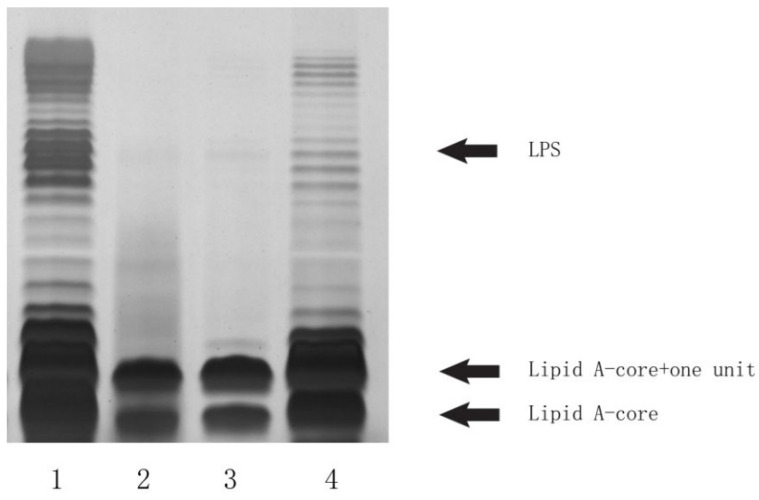
Lipopolysaccharide profiles of LL004 and its derivatives. The extracts were electrophoresed on 12% SDS-PAGE and visualized by silver staining. Lane 1, LL004; lane 2, LL004Δ*wzy*; lane 3, LL004Δ*wzy*::pTrc99a; and lane 4, LL004Δ*wzy*::pTrc99a-*wzy*.

To confirm the role of LL004 O-AGC in OAg biosynthesis, a deletion and complementation experiment was carried out. As shown in the LPS profile (Figure 7), LL004 exhibited a complete LPS, characterized by a lipid A-core band and additional bands corresponding to O-units. However the *wzy* deleted strain only generated a semi-rough LPS phenotype with only one O-unit substitution to the lipid A-core. Moreover, the mutant could be complemented by the plasmid pTrc99a containing the LL004 *wzy* gene, restoring the complete LPS phenotype. These results indicate that the O-AGC characterized by us is involved in LL004 OAg biosynthesis, and that the LL004 OAg is translocated and polymerized by the Wzx/Wzy dependent pathway.

## 3. Materials and Methods

### 3.1. LPS and O-Specific Polysaccharide Extraction

Bacteria were grown to late log phase in 8 L of LB media under constant aeration at 37 °C and pH 7.0. Bacterial cells were washed and dried as described [23] and the LPS was isolated from dried cells by the phenol-water method [24]. The bacterial cells were extracted by stirring under 120 rpm with 50% aqueous phenol for 30 min at 65 °C. The water phase collected by low-speed centrifugation (4000 rpm, 30 min, 4 °C) was dialyzed in distilled water until free from phenol. The dialyzate was lyophilized and dissolved in distilled water. The aqueous solution was treated sequentially with deoxyribonuclease, ribonuclease, and protease K, followed by ultracentrifugation at 8000 rpm for 30 min at 4 °C. The supernatant was then extracted with 50% aqueous phenol, followed by dialysis and lyophilisation to give the LPS. The extracted LPS was bathed in 2% (*v*/*v*) acetic acid in the quantity of 2 mg/mL at 100 °C for 3 h. The precipitated lipid A was removed by freezing ultracentrifugation (13,000× *g*, 30 min, 4 °C). Then, the O-specific polysaccharide was obtained after purification on Sephadex G-50 column with 0.05 M pyridine acetate buffer (pH 4.5).

The molecular weight of the O-specific polysaccharide was determined by high-performance size-exclusion chromatography (HPSEC) [24]. Waters 1525 HPLC equipped with a Ultrahydrogel Linear (7.8 mm × 30.0 cm) column was used to analyze the polysaccharide. A solution of 0.1 mol/L NaNO_3_ was used as the mobile phase and the flow rate was kept at 0.5 mL/min. The eluent was monitored by a Waters 2410 refractive index detector. The column temperature was kept at 40 °C. The concentration of polysaccharide test solution was 5 mg/mL in the mobile phase and the injection volume was 50 μL. Five dextran standards (Mw 2.70, 9.75, 135.03, 300.60, and 2000 kDa) and glucose (Mw 180) were used to plot the calibration standards.

An O-specific polysaccharide sample was treated with 12% aq ammonia at 50 °C for 6 h, ammonia was flushed out, and the following lyophilization [25] afforded the *O*-deacetylated polysaccharide.

### 3.2. Monosaccharide Analysis

The *O*-deacetylated polysaccharide was hydrolyzed with 2 M trifluoroacetic acid (120 °C, 2 h). The extra acid was completely removed by adding methanol with rotary evaporation [25]. The monosaccharides were analysed by high-performance anion-exchange chromatography coupled with pulsed amperometric detection (HPAEC–PAD) method [26]. HPAEC–PAD analysis was performed using an ICS-5000+ ion chromatography system that consisted of a quaternary pump, temperature-controlled column manager, and ED5000 PAD electrochemical cell which consisted of an Au working electrode and pH-Ag/AgCl reference electrode. (Thermo Fisher Scientific, Waltham, MA, USA). The column was a Dionex CarboPac PA20 anion-exchange column including an analytical column (3 × 150 mm) and guard column (3 × 50 mm). The column temperature was 30 °C. The injection volume was 25 μL. The mobile phase consisted of 5 mmol/L sodium hydroxide (NaOH) solution (solvent A) and 5 mmol/L NaOH solution containing 250 mmol/L sodium acetate (NaOAc) (solvent B). The gradient elution condition was as follows: 100% A (0–15 min); 80–0% A, 20–100% B (15–24 min); 100% A (24–35 min). The flow rate was 0.5 mL/min. The monosaccharide types were identified according to the retention time corresponding to the standard monosaccharides.

### 3.3. NMR Analysis

The NMR spectroscopy sample was deuterium-exchanged by freeze-drying from 99.9% D_2_O and then examined as solutions in 99.95% D_2_O. NMR spectra were recorded on a Bruker Ascend 600 MHz spectrometer (Bremen, Germany) at 27 °C. 2D NMR spectra were obtained using standard Bruker software, and the Bruker TopSpin 2.1 program was used to acquire and process the NMR data. For the ^1^H NMR experiment, the pulse program was zg30. For the ^13^C NMR experiment, the pulse program was zgpg30. For the ^31^P NMR experiment, the pulse program was zgpg30. For the ^1^H-^1^H COSY experiment, the pulse program was cosygpmfqf. For the ^13^C-edited HSQC experiment, the pulse program was hsqcedetgpsisp2.3. For the coupled HSQC experiment, the pulse program was hsqcedetgpsisp2.3. For the ^1^H-^13^C HMBC experiment, the pulse program was hmbcgpndqf.

### 3.4. Bacterial Strains, Plasmids, and Growth Conditions

Bacterial strains and plasmids used in this study are summarized in Table S1. The gene-deleted strain was constructed using a λ Red recombinase system, as previously described [27]. Briefly, the plasmid pKD46 was electroporated into the wide-type strain LL004 to enable a direct homologous recombination with PCR products. Then, the target gene was replaced by a kanamycin resistance cassette cloned from plasmid pKD4, after which the kanamycin resistance cassette was subsequently eliminated using pCP20 plasmid. The resultant mutant strain was verified by PCR amplification and sequencing. The complementary strain was constructed by cloning the target gene into plasmid pTrc99a, and the resulting construct was introduced into the corresponding mutant strain. All strains were grown overnight at 37 °C in LB broth for experiments. As required, antibiotics were added at the following final concentrations: ampicillin (Ap), 100 μg/mL; and kanamycin (Km), 50 μg/mL.

### 3.5. Genome Sequencing and Annotation

Genomic DNA used for sequencing was extracted from 1.5 mL of overnight LL004 culture using a DNA extraction kit (Tiangen, China). Genome sequencing and assembly was performed by Novogene Co., Ltd. (Beijing, China). Then, we utilized Artemis [28] to annotate genes, BLAST and PSI-BLAST [29] to search genes and proteins against the available databases including GenBank (www.ncbi.nlm.nih.gov/genbank, accessed on 24 November 2021) and the Pfam protein families database (pfam.sanger.ac.uk, accessed on 24 November 2021), and TMHMM v2.0 (www.cbs.dtu.dk/services/TMHMM-2.0/, accessed on 24 November 2021) to identity potential transmembrane domains within proteins. Finally, the *wcaM* to *hisI* region was retrieved for further analysis. The O-AGC sequence of LL004 was deposited to Genbank under accession number OK073909.

### 3.6. SDS-PAGE Analysis of LPS

LPS used for sodium dodecyl sulphate polyacrylamide gel electrophoresis (SDS-PAGE) analysis was prepared using the hot aqueous-phenol method, as previously described [30]. The extracted LPS were separated using 12% SDS-PAGE at 50 V for 30 min and 100 V for 2 h, and, subsequently, they were visualized by silver staining using the Fast Silver Stain Kit (no. P0017S, Beyotime, Shanghai, China), according to the manufacturer’s protocol. The gel image was captured using a GS900 Calibrated Densitometer (BioRad Laboratories, Hercules, CA, USA).

## 4. Conclusions

In this study, we elucidated the O-antigen structure of an *E. coli* strain LL004, which is composed of a tetrasaccharide repeating unit, and which has not been reported in any *E. coli* serogroups. We also defined the O-antigen gene cluster by genomic analysis and confirmed its role in OAg biosynthesis experimentally. Our results indicate that LL004 should be a novel serogroup of *E. coli*. Thus far, *E. coli* O serogroups from O1 to O188 have been internationally recognized by the International Centre for Reference and Research located at the Statens Serum Institut (SSI) in Denmark [31]. Therefore, combining our genetic and structural data, we suggest that LL004 be designated as O189 in numerical order.

## Figures and Tables

**Table 1:** ^1^H and ^13^C NMR chemical shifts of the O-specific polysaccharide from *E. coli* strain LL004.

Residue	Chemical Shifts (ppm)							
	1-H/1-C	2-H/2-C	3-H/3-C	4-H/4-C	5-H/5-C	6-H/6-C	NAc (C=O)	OAc (C=O)
**A** β-d-Gal*p*A ^1^*J*_H1,C1_ 160.3 Hz	4.71 (^3^*J*_H,H_ 6.3 Hz)/102.7	3.55 (^3^*J*_H,H_ 11.0, 6.3 Hz)/69.1	3.52 (^3^*J*_H,H_ 11.0 Hz)/80.4	4.47 (singlet), 4.50 (singlet)/69.6	4.27/76.9	-/175.0	-	
**B** β-d-Gal*p*NAc ^1^*J*_H1,C1_ 165.2 Hz	4.60 (^3^*J*_H,H_ 8.9 Hz)/102.6	3.95 (^3^*J*_H,H_ 8.9, 8.9 Hz)/51.4	3.80 (^3^*J*_H,H_ 8.9 Hz)/79.6	4.12 (singlet)/68.1	3.63/72.3	3.69/60.8	1.94/22.4 (174.9)	
**C**(**D**) β-d-Gal*p* ^1^*J*_H1,C1_ 160.2 Hz	4.38 (^3^*J*_H,H_ 6.2 Hz)/103.0, 4.30 (^3^*J*_H,H_ 6.4 Hz)/103.3	3.31 (^3^*J*_H,H_ 9.0, 6.4 Hz)/71.1	3.67 (^3^*J*_H,H_ 9.0 Hz)/72.3	4.02 (singlet)/76.3	3.62/74.7	3.69/60.8	-	-
**E** β-d-Glc*p*NAc6OAc ^1^*J*_H1,C1_ 165.2 Hz	4.61 (^3^*J*_H,H_ 11.0 Hz)/102.6	3.72 (^3^*J*_H,H_ 11.0, 11.0 Hz)/55.1	3.64 (^3^*J*_H,H_ 11.0 Hz)/79.0	3.65/72.5	3.73/72.1	4.44, 4.31/62.8	1.94/22.4 (174.9)	2.07/20.3 (174.0)

**Table 2:** ^1^H and ^13^C NMR chemical shifts of the *O*-deacetylated O-specific polysaccharide from *E. coli* strain LL004.

Residue	Chemical Shifts (ppm)						
	1-H/1-C	2-H/2-C	3-H/3-C	4-H/4-C	5-H/5-C	6-H/6-C	NAc (C=O)
**A** β-d-Gal*p*A	4.70 (^3^*J*_H,H_ 6.6 Hz)/102.6	3.51 (^3^*J*_H,H_ 10.8, 6.6 Hz)/69.0	3.48/80.2	4.49 (singlet)/69.7	4.21 (singlet)/77.2	-/175.0	-
**B** β-d-Gal*p*NAc	4.57 (^3^*J*_H,H_ 9.3 Hz)/02.5	3.94 (^3^*J*_H,H_ 9.3, 9.3 Hz)/51.4	3.79 (^3^*J*_H,H_ 9.3 Hz)/79.5	4.11 (singlet)/68.0	3.62/72.2	3.69/60.8	1.92/22.2 (175.0)
**C**(**D**) β-d-Gal*p*	4.37 (^3^*J*_H,H_ 8.6 Hz)/103.0	3.29 (^3^*J*_H,H_ 8.6, 8.6 Hz)/70.9	3.67 (^3^*J*_H,H_ 8.6 Hz)/72.4	4.00 (singlet)/76.3	3.61/74.5	3.69/60.8	-
**E** β-d-Glc*p*NAc	4.57 (^3^*J*_H,H_ 11.5 Hz)/102.5	3.70 (^3^*J*_H,H_ 11.5, 8.5 Hz)/54.9	3.66/74.8	3.61/74.6	3.49/74.6	3.86, 3.75/59.9	1.93/22.2 (175.0)

**Table 3:** Characteristics of the ORFs in LL004 O-antigen gene cluster.

Orf No.	Gene Name	Position of Gene	G+C Content (%)	Similar Protein(s), Strain(s) (Genbank Accession No.)	%Identical/%Similar (Total No. of aa)	Putative Function of Protein
	*wcaM*	1.1394	47.31	colanic acid biosynthesis protein WcaM [*Escherichia coli*] (EEU3037011.1)	99/99 (464)	colanic acid biosynthesis protein
1	*gnu*	1553.2548	49.68	*N*-acetyl-alpha-d-glucosaminyl-diphospho-ditrans,octacis-undecaprenol 4-epimerase [*Escherichia coli*] (WP_113419175.1)	99/100 (331)	*N*-acetyl-alpha-d-glucosaminyl-diphospho-ditrans, octacis-undecaprenol 4-epimerase
2	*galF*	2791.3684	50.78	UTP--glucose-1-phosphate uridylyltransferase GalF [*Escherichia coli*] (WP_113415284.1)	99/100 (297)	UTP--glucose-1-phosphate uridylyltransferase
3	*wzx*	4151.5272	28.6	oligosaccharide flippase family protein [*Aeromonas sp.* 1419](WP_216951369.1)	37/60 (395)	flippase
4	*wzy*	5259.6449	28.88	Wzy [*Proteus mirabilis*] (AXY99601.1)	32/49 (394)	polymerase
5	*gtr1*	6439.7191	24.7	glycosyltransferase family 25 protein [*Escherichia coli*] (WP_001570044.1)	100/100 (250)	glycosyltransferase
6	*gtr2*	7193.8089	28.65	glycosyltransferase family 2 protein [*Escherichia coli*] (WP_000856120.1)	100/100 (298)	glycosyltransferase
7	*orf7*	8100.9362	31.03	hypothetical protein [*Escherichia coli*] (WP_152696934.1)	99/100 (420)	hypothetical protein
8	*gtr3*	9366.10184	33.82	glycosyltransferase [*Escherichia coli*](WP_001285383.1)	100/100 (272)	beta-1,3-galactosyltransferase
9	*gnd*	10271.11677	49.68	6-phosphogluconate dehydrogenase, decarboxylating [*Klebsiella pneumoniae* IS22] (CDK78112.1)	99/100 (468)	6-phosphogluconate dehydrogenase, decarboxylating
10	*ugd*	11926.13092	42.93	UDP-glucose 6-dehydrogenase [*Escherichia coli*] (BAV90487.1)	99/100 (397)	UDP-glucose 6-dehydrogenase
11	*gne*	complement (13158.14162)	42.68	NAD-dependent epimerase [*Escherichia coli*] (WP_063625168.1)	99/100 (334)	UDP-N-acetylglucosamine 4-epimerase
12	*wzz*	14571.15548	46.72	chain length determinant protein [*Escherichia coli* 907357](ESA76081.1)	99/99 (344)	Chain length determinant protein
	*hisI*	complement (15644.16255)	53.1	bifunctional phosphoribosyl-AMP cyclohydrolase/phosphoribosyl-ATP diphosphatase HisIE [*Escherichia coli*](WP_000954885.1)	100/100 (203)	Phosphoribosyl-AMP cyclohydrolase

## Data Availability

Genkbank accession number: OK073909.

## Supplementary Materials

The following are available online at https://www.mdpi.com/article/10.3390/ijms222312746/s1.
