# Supplementary figures and images for: Structural Determination and Genetic Identification of the O-Antigen from an Escherichia coli Strain, LL004, Representing a Novel Serogroup
Source: Int J Mol Sci. 2021 Nov 25;22(23):12746. doi: 10.3390/ijms222312746 (PMC8657804; doi:10.3390/ijms222312746)

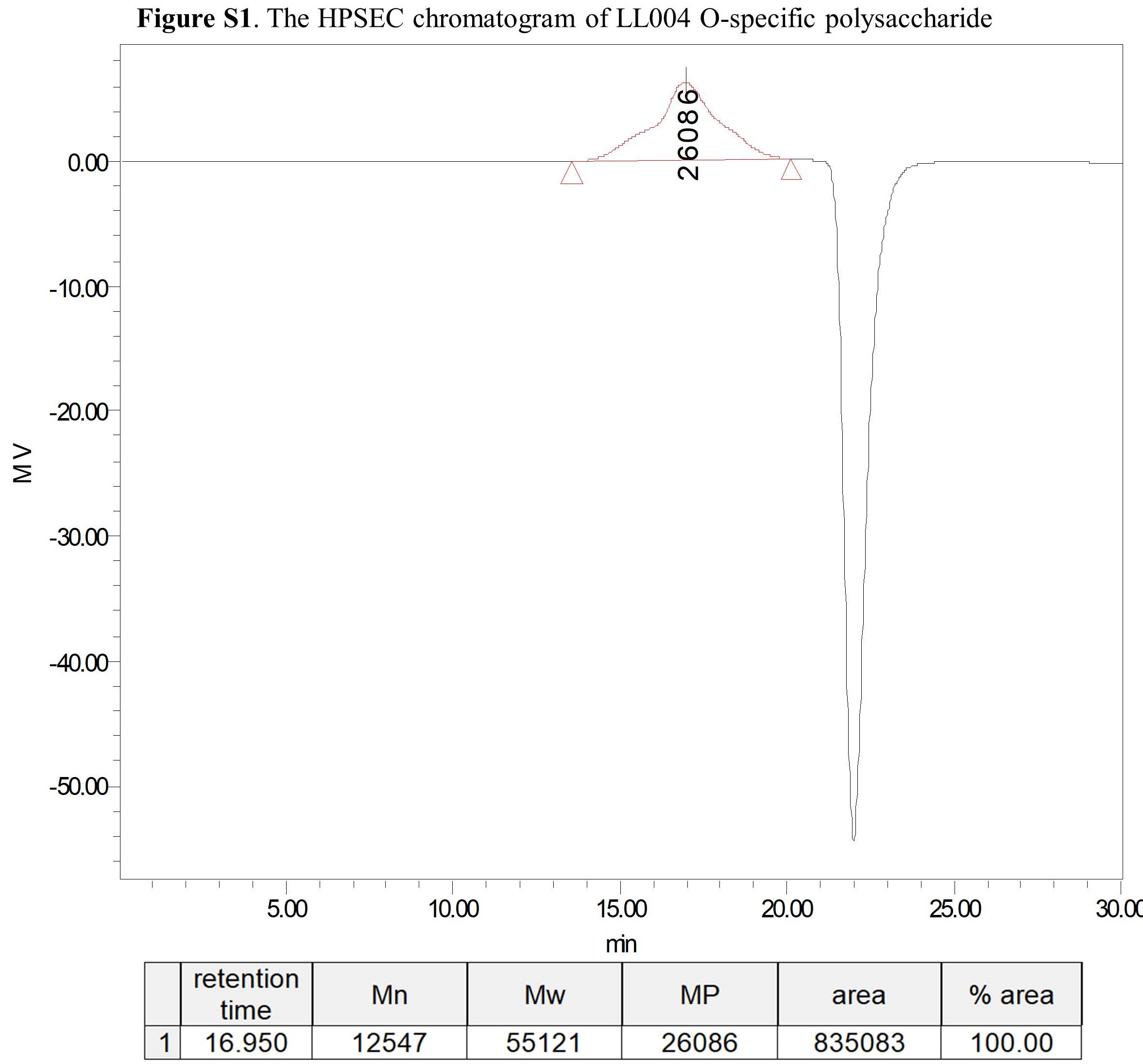

Supplement: Supplementary file 1 [file ijms-22-12746-s001.zip › ijms-1395827-supplementary/pictures for SI-LL004/Figure S1.jpg]

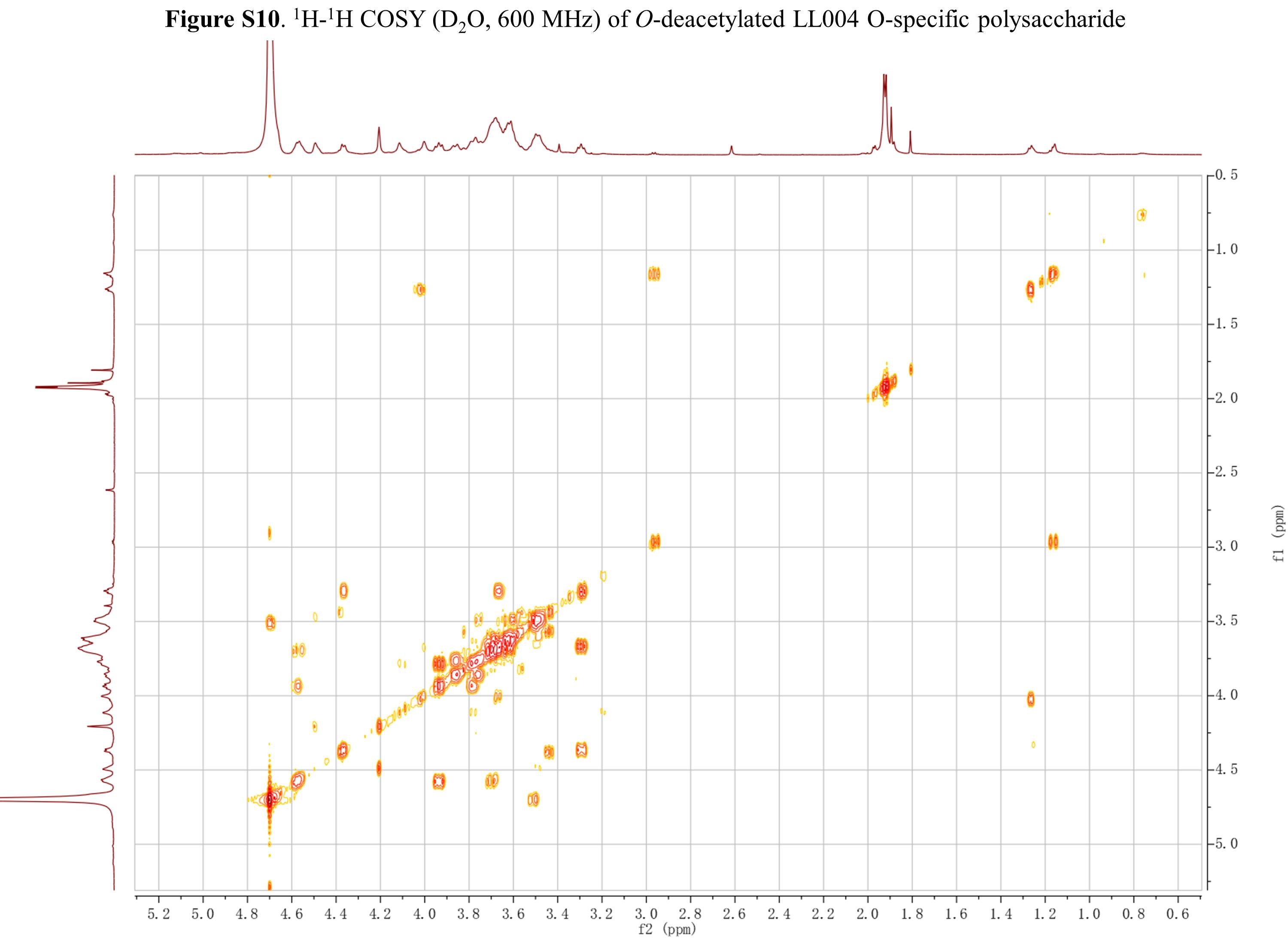

Supplement: Supplementary file 1 [file ijms-22-12746-s001.zip › ijms-1395827-supplementary/pictures for SI-LL004/Figure S10.jpg]

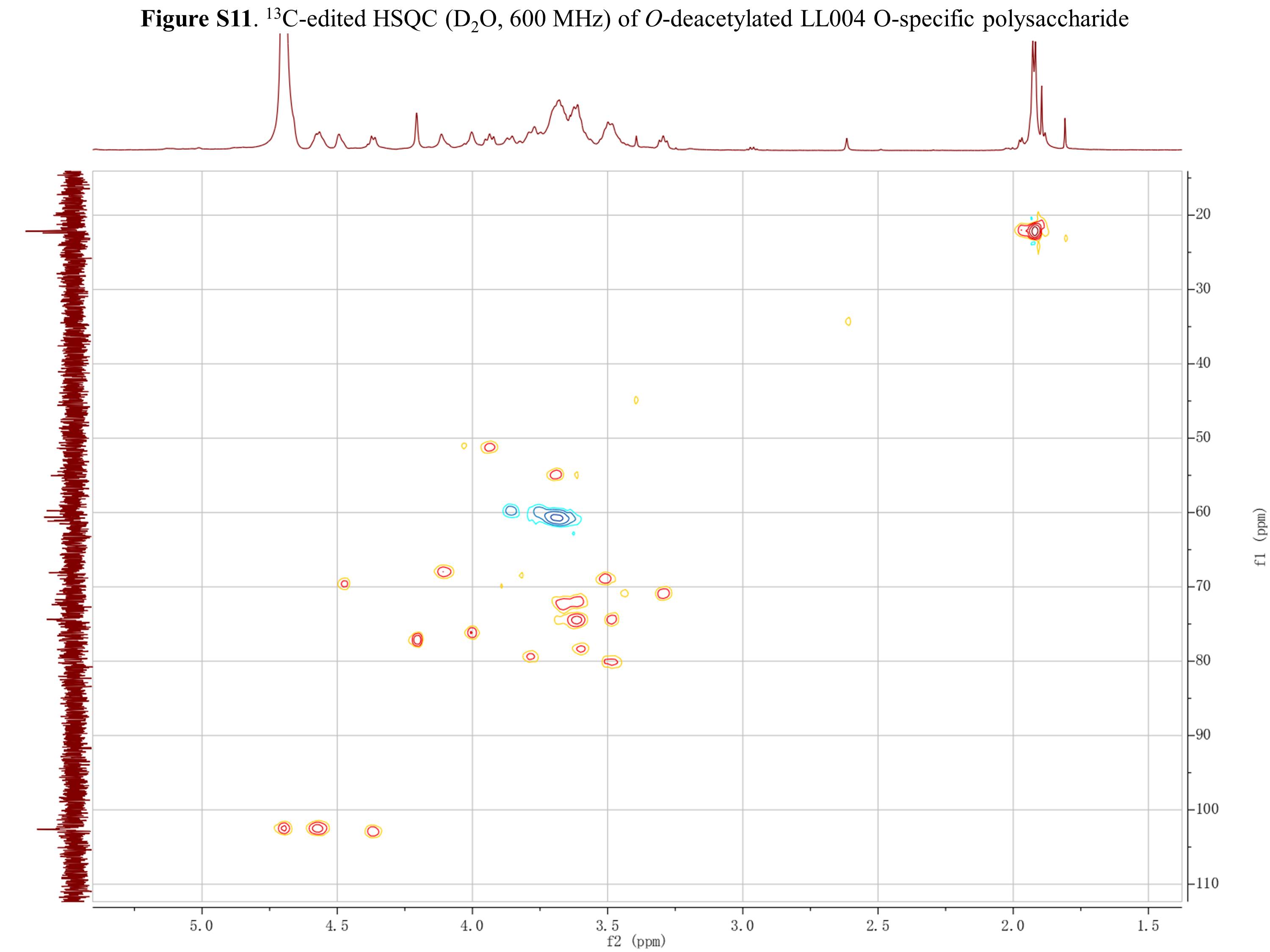

Supplement: Supplementary file 1 [file ijms-22-12746-s001.zip › ijms-1395827-supplementary/pictures for SI-LL004/Figure S11.jpg]

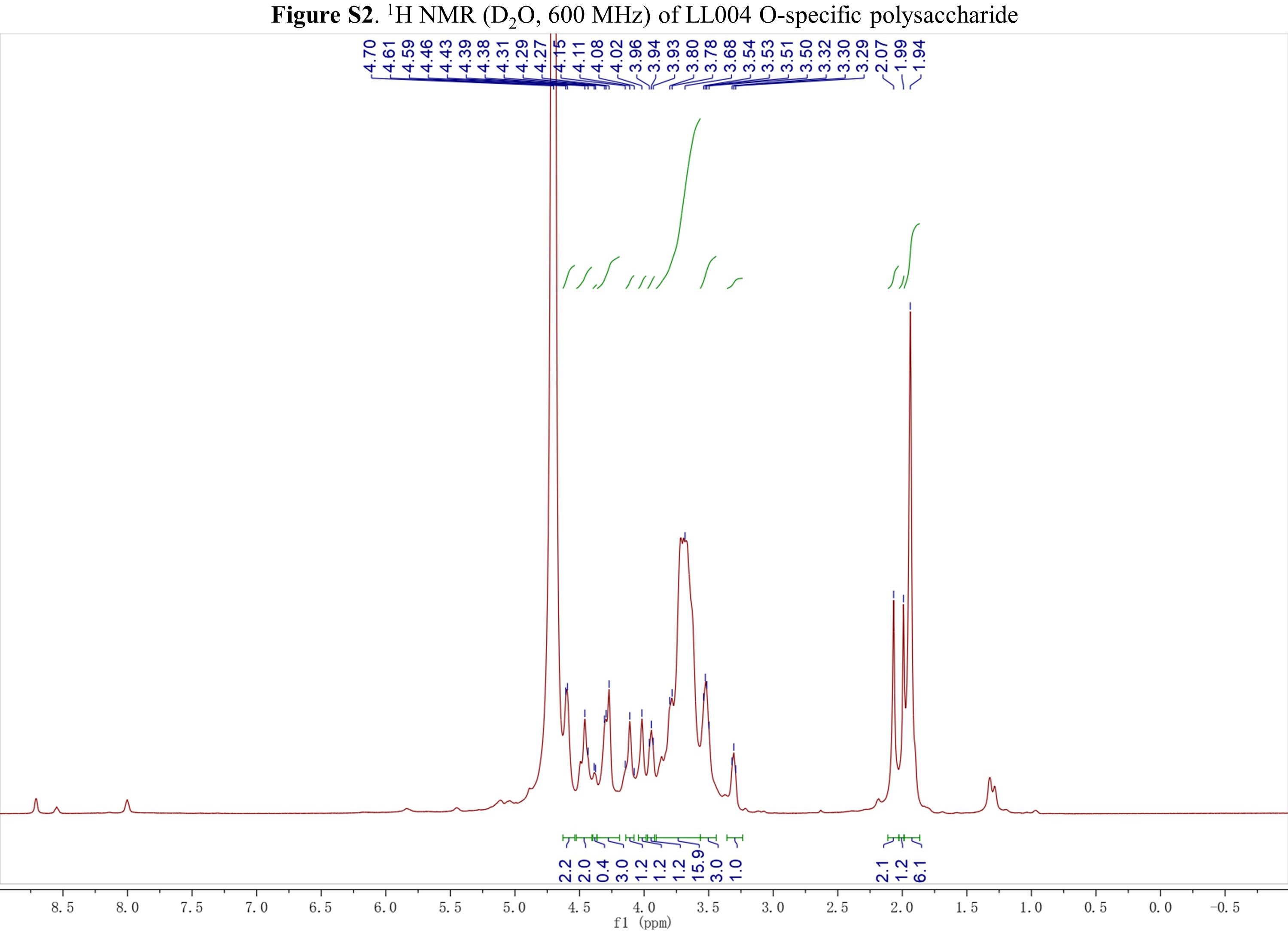

Supplement: Supplementary file 1 [file ijms-22-12746-s001.zip › ijms-1395827-supplementary/pictures for SI-LL004/Figure S2.jpg]

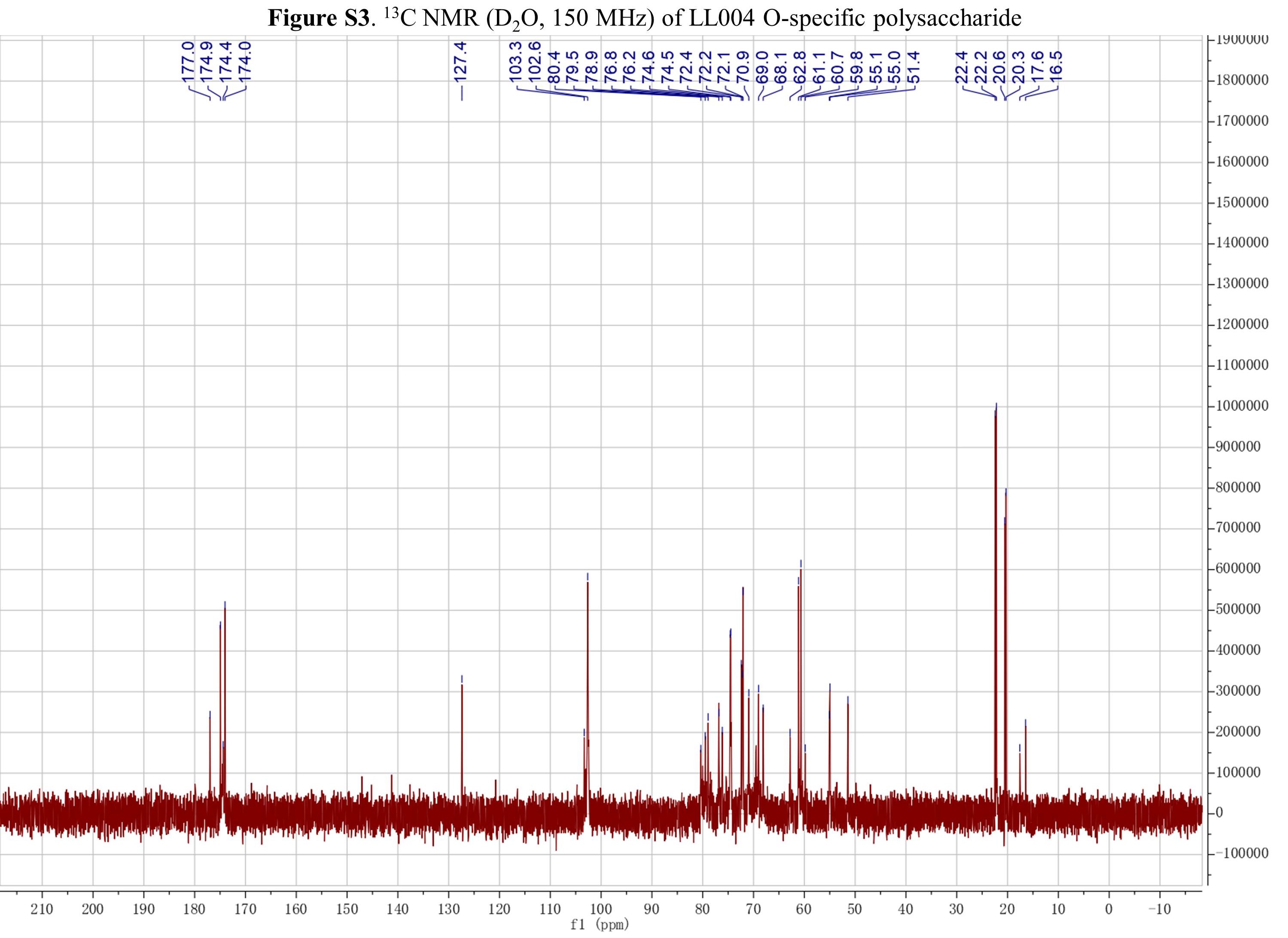

Supplement: Supplementary file 1 [file ijms-22-12746-s001.zip › ijms-1395827-supplementary/pictures for SI-LL004/Figure S3.jpg]

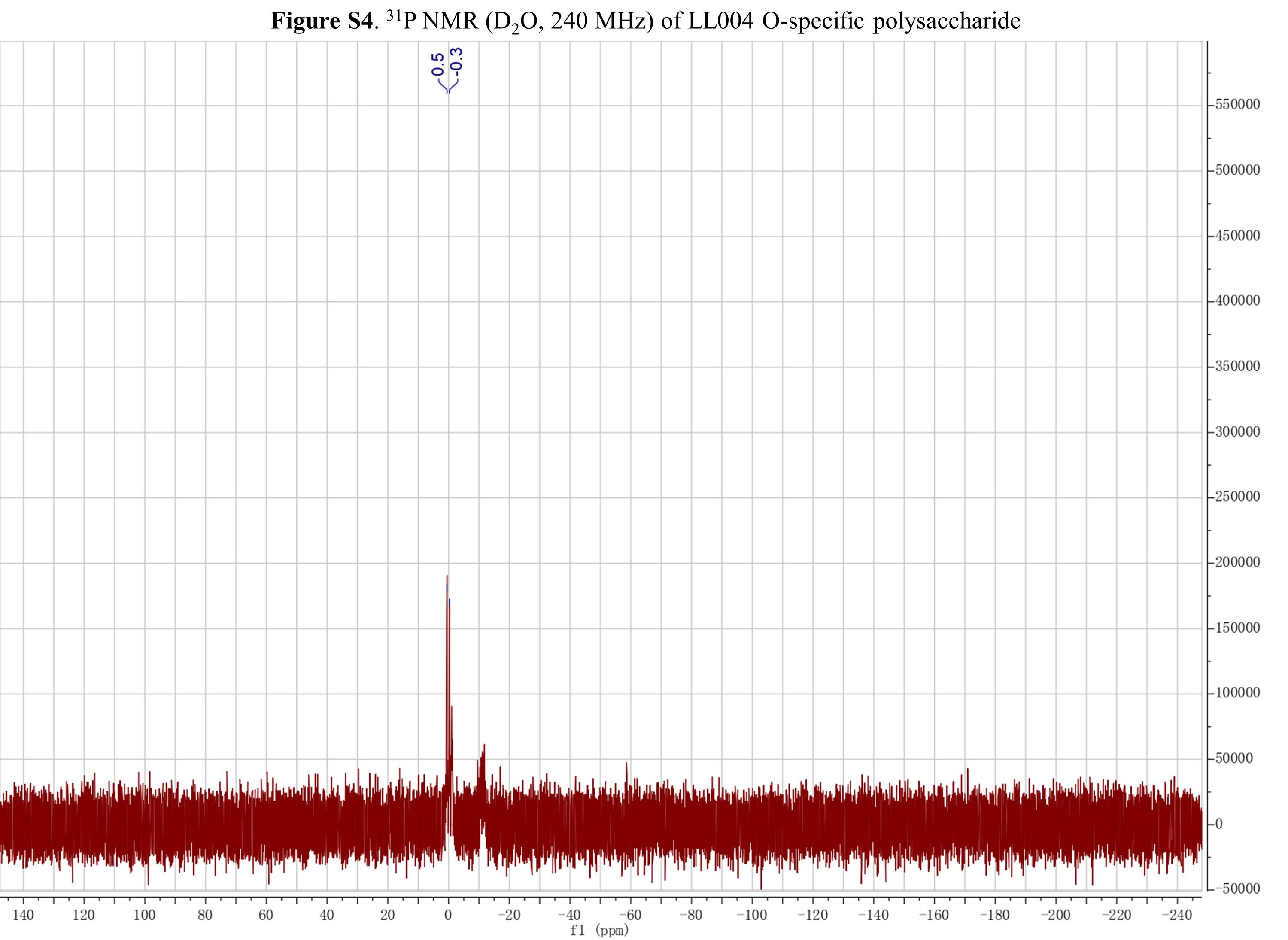

Supplement: Supplementary file 1 [file ijms-22-12746-s001.zip › ijms-1395827-supplementary/pictures for SI-LL004/Figure S4.jpg]

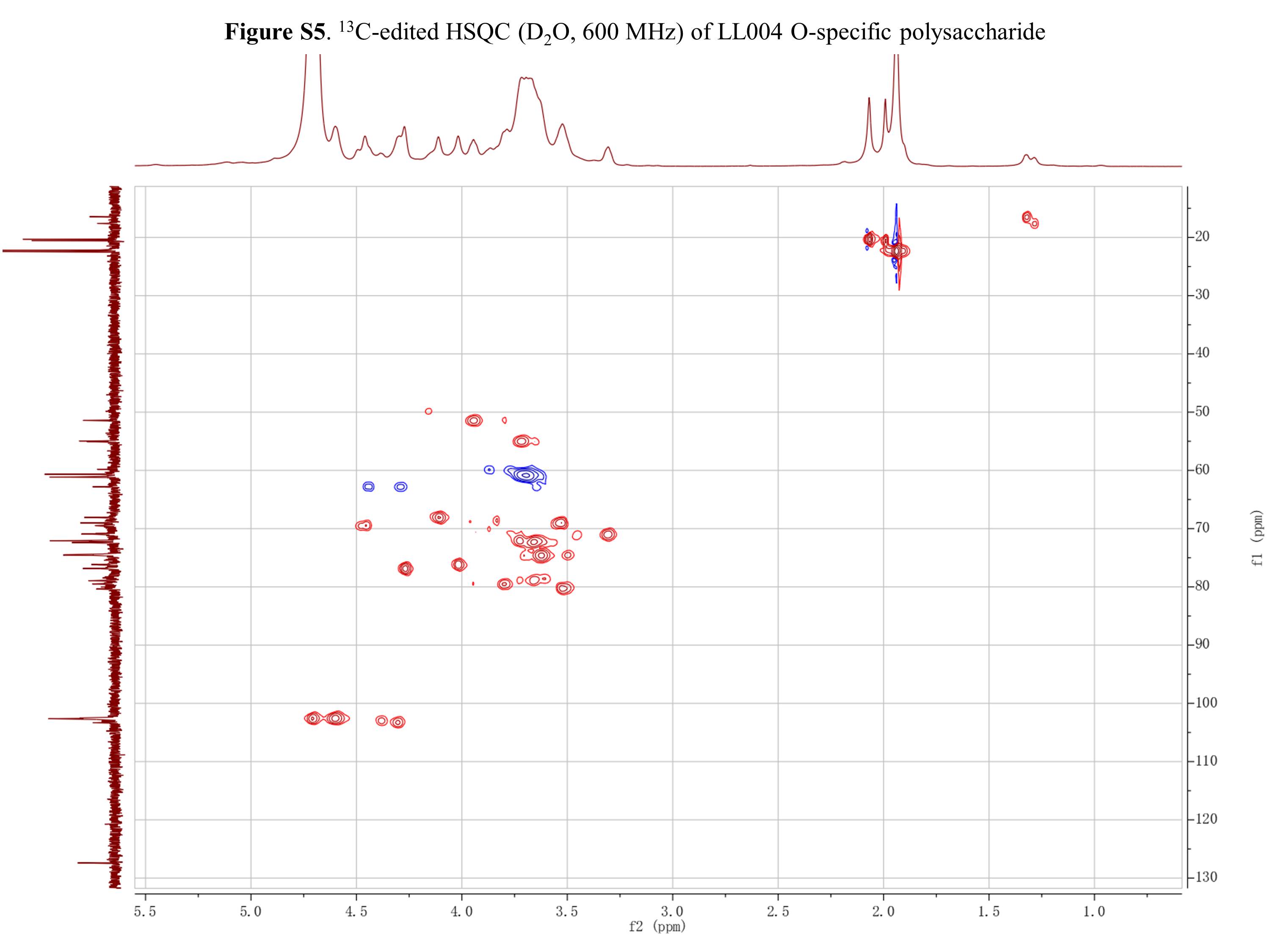

Supplement: Supplementary file 1 [file ijms-22-12746-s001.zip › ijms-1395827-supplementary/pictures for SI-LL004/Figure S5.jpg]

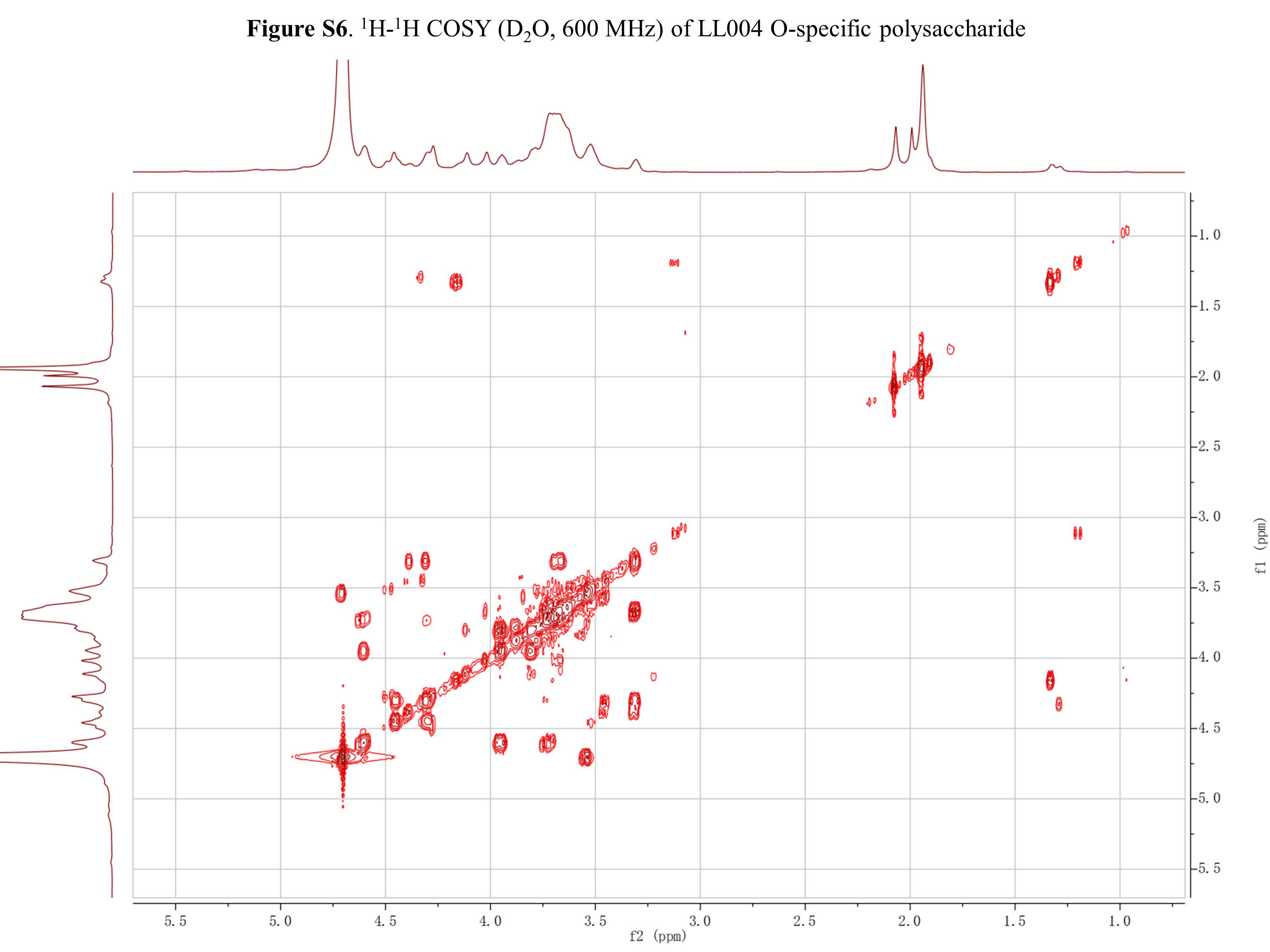

Supplement: Supplementary file 1 [file ijms-22-12746-s001.zip › ijms-1395827-supplementary/pictures for SI-LL004/Figure S6.jpg]

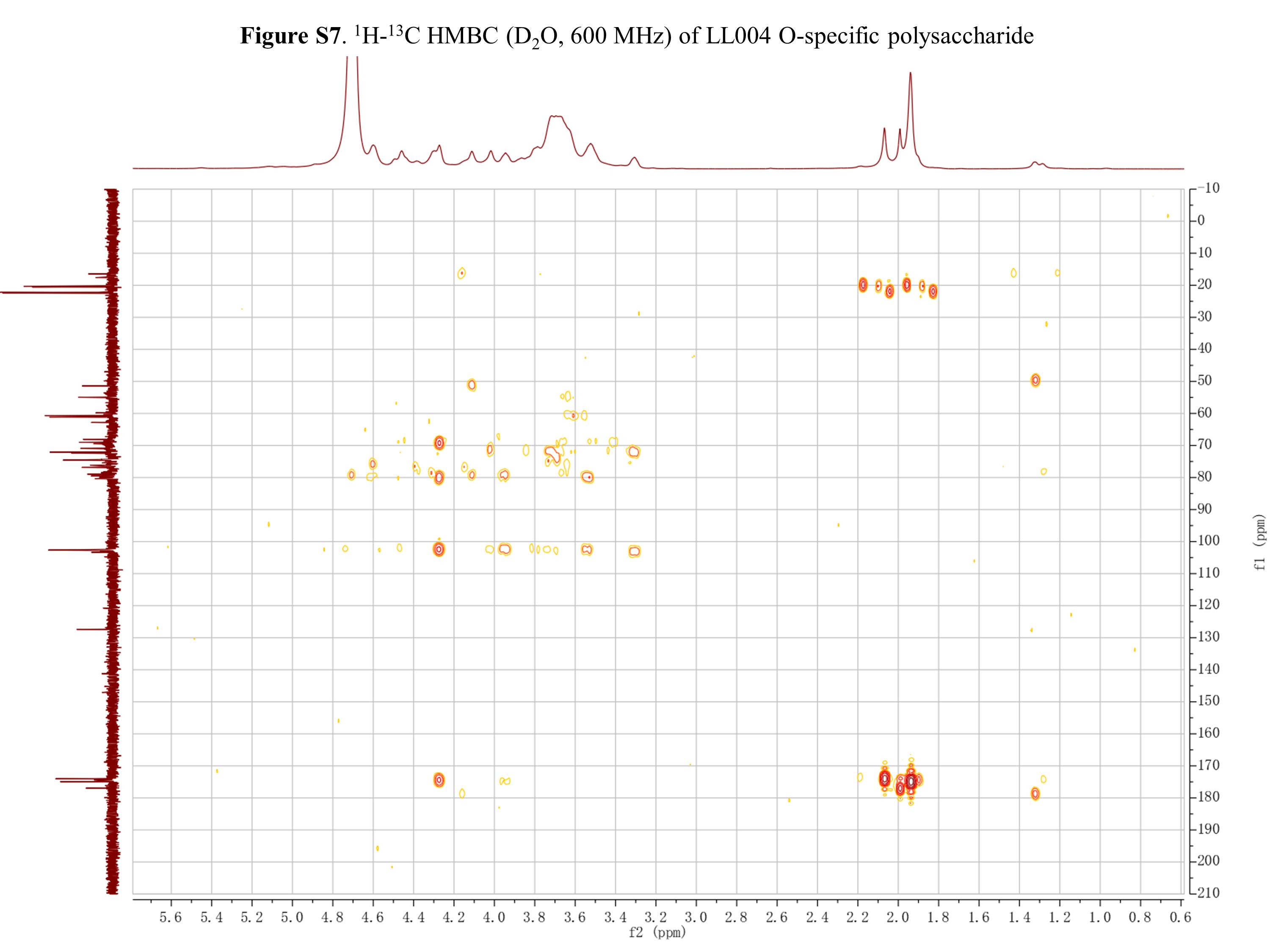

Supplement: Supplementary file 1 [file ijms-22-12746-s001.zip › ijms-1395827-supplementary/pictures for SI-LL004/Figure S7.jpg]

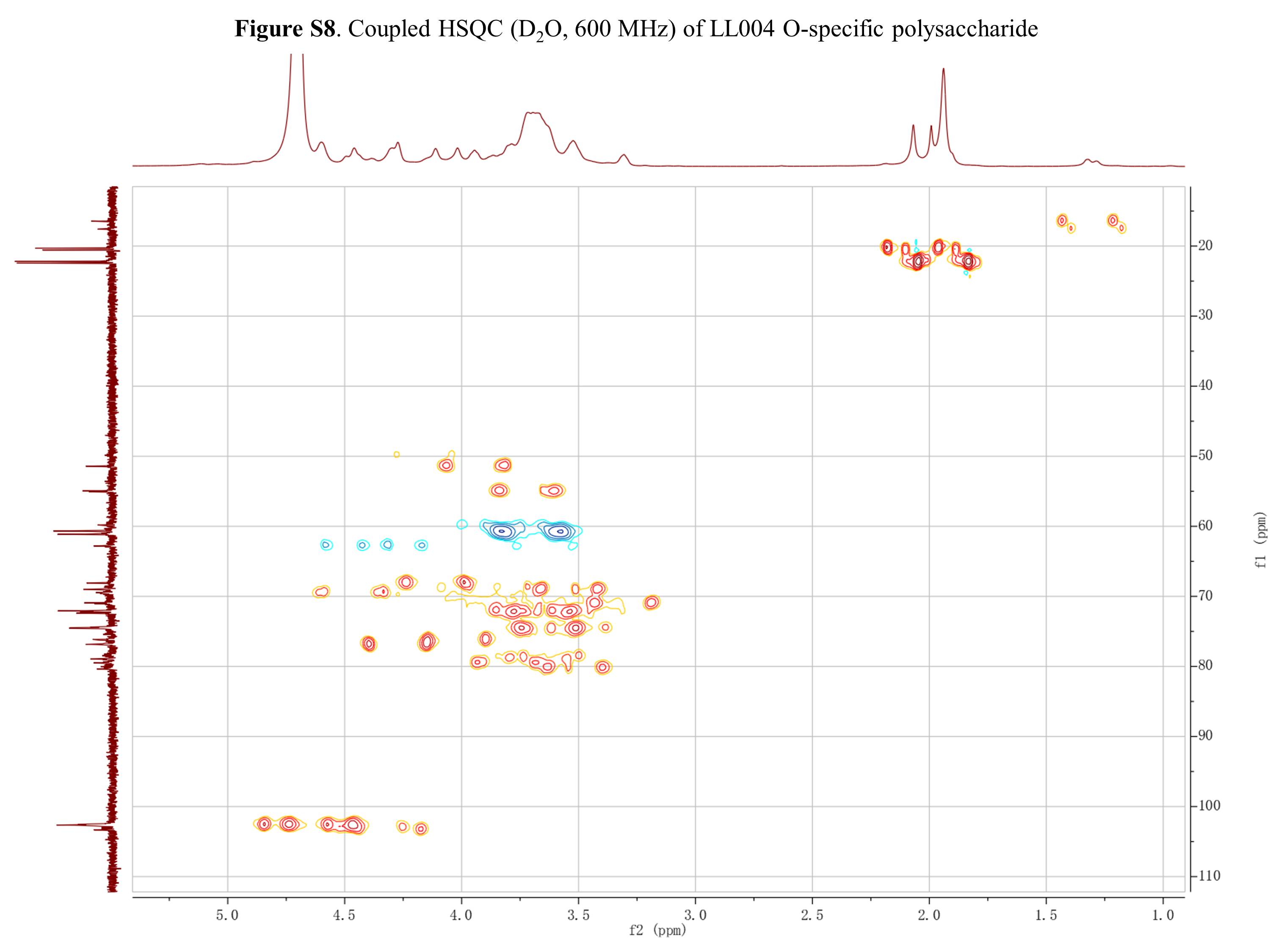

Supplement: Supplementary file 1 [file ijms-22-12746-s001.zip › ijms-1395827-supplementary/pictures for SI-LL004/Figure S8.jpg]

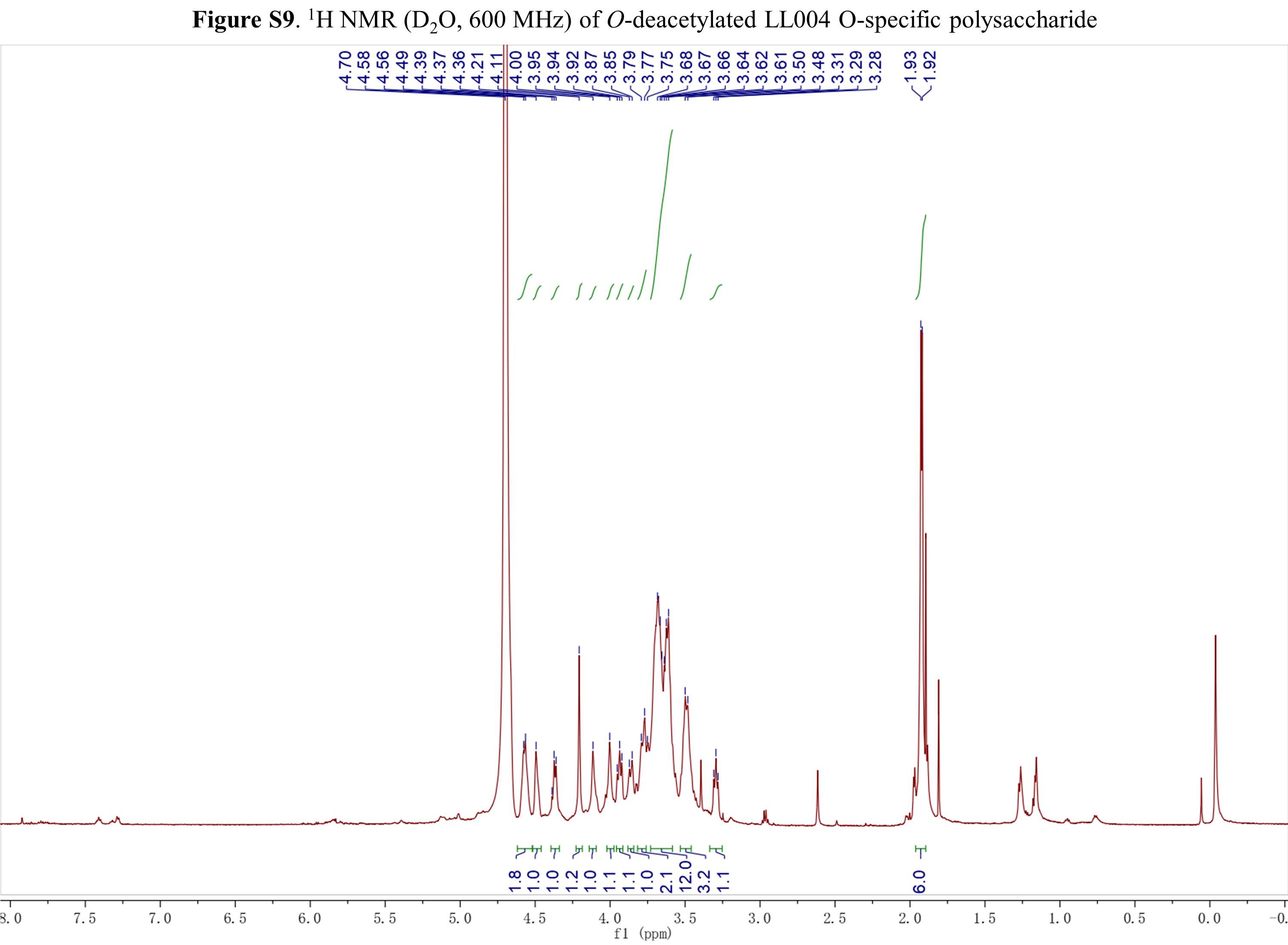

Supplement: Supplementary file 1 [file ijms-22-12746-s001.zip › ijms-1395827-supplementary/pictures for SI-LL004/Figure S9.jpg]
